# Primary hemostasis in fetal growth restricted neonates studied *via* PFA-100 in cord blood samples

**DOI:** 10.3389/fped.2022.946932

**Published:** 2022-09-08

**Authors:** Maria Kollia, Nicoletta Iacovidou, Zoi Iliodromiti, Abraham Pouliakis, Rozeta Sokou, Vasiliki Mougiou, Maria Boutsikou, Marianna Politou, Theodora Boutsikou, Serena Valsami

**Affiliations:** ^1^Neonatal Department, Medical School, National and Kapodistrian University of Athens, Athens, Greece; ^2^2nd Department of Pathology, “Attikon” University Hospital, National and Kapodistrian University of Athens, Athens, Greece; ^3^Haematology Laboratory-Blood Bank, Aretaieio Hospital, National and Kapodistrian University of Athens, Athens, Greece

**Keywords:** FGR, PFA-100, closure time, platelets, prematurity

## Abstract

**Background:**

Platelet function of fetal growth restricted (FGR) neonates remains a field of debate. Platelet function analyzer (PFA-100) offers a quantitative *in vitro* assessment of primary, platelet-related hemostasis. Our aim was to examine platelet function using PFA-100 in FGR neonates and associate our results with perinatal parameters.

**Methods:**

PFA-100 was applied on 74 FGR neonates, 48 full-term (>37 weeks' gestation) and 26 preterm neonates (<37 weeks). The control group consisted of 118 healthy neonates. Two closure times (CTs) with COL/EPI and COL/ADP cartridges were determined on cord blood samples for each subject. Statistical analysis was performed by SAS 9.4. The statistical significance level was set at 0.05 and all tests were two-tailed.

**Results:**

COL/EPI CTs were prolonged in FGR (median 132 s, IQR 95–181 s) compared with control neonates (median 112.5 s, IQR 93–145 s), *p* = 0.04. Median COL/EPI CT for term and preterm FGR neonates was 126 s (IQR 90–157 s) and 137 s (IQR 104–203), respectively (*p* = 0.001), and COL/ADP CT was 70 s (IQR 62–80 s) for term and 75 s (IQR 68–82 s) for preterm FGR neonates (*p* = 0.08). Among FGR neonates, COL/EPI CT was related with delivery time (with preterm neonates exhibiting prolonged COL/EPI CTs), *p* = 0.05. No correlation was proved between both CTs and hematological parameters in FGR neonates.

**Conclusion:**

FGR neonates showed impaired platelet function *via* PFA-100, with preterm FGR neonates confronting the greatest risk. Prolonged COL/EPI CTs in FGR neonates seemed to be independent of hematological parameters and could warn for closer evaluation during the first days of their lives.

## Introduction

Fetal growth restriction (FGR), formerly known as intrauterine growth restriction (IUGR), refers to the failure of fetus to reach its intrinsic growth potential, due to pathological causes of maternal, fetal, placental, or genetic origin (1–3). FGR affects 5–10% of all pregnancies (4), is one of the leading causes of perinatal morbidity and mortality, and is associated with long-term chronic diseases (3, 5, 6). A main characteristic of FGR neonates is thrombocytopenia (7–9). Thrombocytopenia could be attributed to destruction of platelets caused by placental vascular pathology (10), shunt of stem cells to erythropoiesis due to intrauterine hypoxia (11), or immaturity of liver and spleen due to redistribution of blood flow and the brain-sparing effect. Thrombocytopenia of FGR neonates is independently associated with lower gestational age (GA) at birth (12).

The investigation of neonatal platelet function in FGR neonates remains an issue of ongoing research with conflicting results so far. Platelet function studies concern mainly neonates born to mothers with pregnancy-induced hypertension (PIH), showing decreased platelet adhesion (13) and expression of glycoproteins on surfaces of activated platelets (14). On the contrary, flow cytometry studies reported higher *in vitro* responsiveness of neonatal platelets to various agonists (15). Regarding preterm FGR neonates, data examining platelet reactivity are scarce, with platelets from preterm offsprings of PIH-pregnancies displaying lower platelet adhesion on cone and platelet analyzer (CPA) (13).

In this study, we hypothesized that FGR neonates have a distinct platelet function, possibly affecting bleeding parameters and subsequent thrombotic risk. Thus, we aimed to examine platelet responsiveness using platelet function analyzer (PFA-100) in this group of neonates and associate our results with several perinatal parameters.

## Methods

This is a prospective cohort study of full-term and preterm FGR neonates born at Aretaieio Hospital, National and Kapodistrian University of Athens, during a 2-year study period (January 2017–December 2018). The Hospital Ethics Review Committee approved the study and mothers signed an informed consent prior to recruitment. Seventy-four FGR neonates, 48 full-term (>37 weeks' gestation) and 26 preterm neonates (<37 weeks' gestation), were included. Control group consisted of 118 appropriate for gestational age (AGA) neonates, 104 full-term and 14 preterm, who were previously published by our research team (16). Control and FGR group samples were collected during the same study period, at the same hospital by the same team of neonatologists.

**Figure 1:**
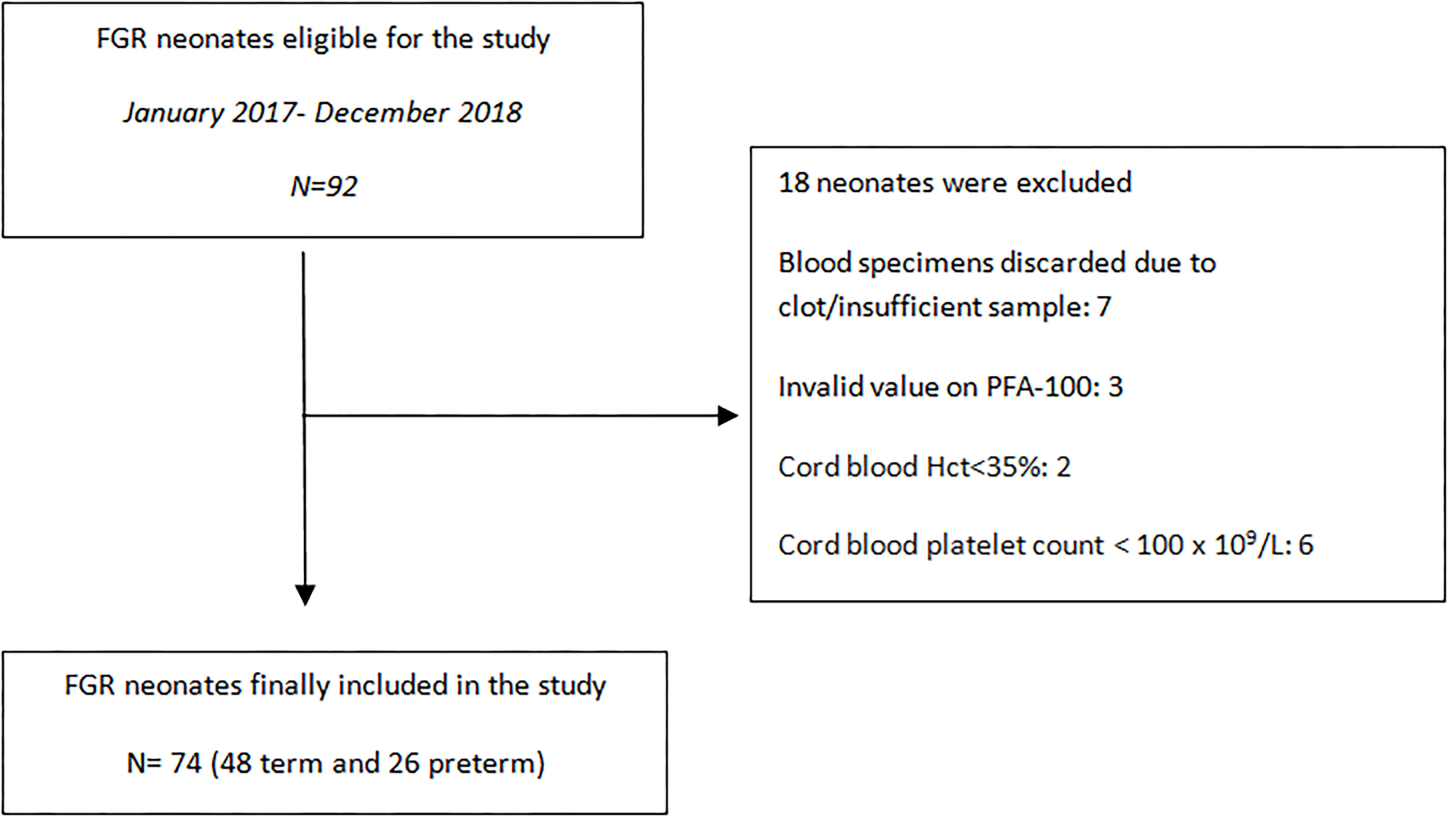
Study population flowchart.

Demographic data and perinatal parameters were listed from the maternal and neonatal medical records and are summarized in Tables 1, 2. Neonates were monitored till discharge and any postnatal complication was recorded. Inclusion criteria for the FGR group consisted of a prenatal diagnosis of estimated fetal weight (EFW) <10th centile and a distinct FGR causative pattern. The AUDIPOG computer-generated program was used to calculate the customized percentile for each pregnancy. Normal birth weight (BW) percentile limits were adjusted using significant determinants of BW (maternal age, height and weight, GA, parity, and gender) (https://www.audipog.net/Estimation-croissance). Intrauterine follow up of FGR fetuses included repeated Doppler studies assessing the pulsatility index (PI) of the uterine, umbilical, and cerebral arteries. A causative FGR pattern refers to abnormal PI values, thrombophilia, hypothyroidism, smoking, PIH/preeclampsia, diabetes (type 1 or gestational), and multiple pregnancy. In 22 cases, PI values were in the upper limits for the corresponding GA indicating compromised fetal perfusion (17, 18). Notably, 16 of 74 FGR neonates were born by twin pregnancies and 10 of them were siblings of 5 dichorionic/diamniotic pregnancies. Additionally, the cephalization index (CI) (ratio of head circumference to body weight) (19) and the HC/AC ratio (ratio of head circumference to abdominal circumference) were also used as measures of fetal compromise (20). Exclusion criteria, for both groups, included cord blood Hct <35%, cord blood platelet count <100 × 10^9^/L, temperature at birth <35°C, evidence of chorioamnionitis, and any major chromosomal anomaly. Cut-off values of Hct and platelets were selected according to PFA-100 manufacturer's instructions. Data regarding recruitment of our study are presented in the flowchart (Figure 1).

**Table 1:** Demographic data of control, FGR, term FGR, and preterm FGR neonates.

**Variable**	**Control**	**FGR**	***P*-value**	**Term FGR**	**Preterm FGR**	***P*-value**
N	118	74		48	26	
GA (weeks)	39^+1^ (38^+2^–39^+6^)	37^+6^ (36^+1^–39^+3^)	**<0.001**	39^+2^ (38–39^+4^)	35^+4^ (34^+1^–36^+2^)	**<0.001**
BW (grams)	3,305 (3,080–3,560)	2,510 (2,080–2,740)	**<0.001**	2,670 (2,510–2,810)	1,895 (1,700–2,190)	**<0.001**
BW centile	50.0 (35.0–69.0)	5.0 (2.0–8.0)	**<0.001**	6.0 (4.5–8.0)	2.0 (2.0–5.0)	**<0.001**
Female (%)	47	54	0.32	48	65	0.15
IVF (%)	5	12	0.08	6	23	**0.03**
CS (%)	66	78	0.07	69	96	**0.006**

Arithmetic data are shown as median values (25–75th pctl). Categorical characteristics are presented as percentages. Bold values indicate statistical significance. GA, gestational age; BW, birthweight; IVF, *in vitro* fertilization; CS, caesarian section.

**Table 2:** Perinatal parameters of control, FGR, term FGR, and preterm FGR neonates.

**Variable**	**Control**	**FGR**	***P*-value**	**Term FGR**	**Preterm FGR**	***P*-value**
N	118	74		48	26	
T at birth (°C)	36.3 (36.1–36.6)	36.10 (35.90–36.40)	**0.007**	36.2 (36.0–36.4)	36.0 (35.8–36.5)	0.0767
APGAR 1': <5*	0	1	**0.04**	0	4	**0.02**
5–7 (%)	0	4		0	12	
8–10 (%)	100	95		100	85	
APGAR 5':8–10*	100	100	–	100	100	–
Blood group: O*	39	36	0.73	35	38	0.80
Neonatal WBC (x10^9^/L)	13.2 (11.4–15.2)	11.4 (9.54–13.5)	**<0.001**	12.8 (10.7–14.6)	9.57 (7.02–11.1)	0.38
Neonatal Hb (g/dl)	15.15 (14.4–16.05)	16.10 (15.10–17.00)	**<0.001**	16.1 (15.1–17.5)	16.2 (15.0–16.9)	0.54
Neonatal Hct (%)	45.1 (42.2–47.75)	48.00 (45.00–50.80)	**<0.001**	48.0 (45.0–53.0)	48.0 (44.7–50.0)	0.58
Neonatal PLT (x10^9^/L)	251.0 (207.5–296.0)	243.0 (187.0–283.0)	0.16	248.0 (187.0–291.0)	233.5 (192.0–277.0)	0.47
Neonatal MPV (fL)	9.8 (8.4–10.5)	9.70 (8.00–10.40)	0.59	9.9 (8.2–10.5)	8.8 (7.6–10.0)	0.11
Aspirin: no*	92	80	**0.04**	81	77	**<0.001**
<7 days before labor	2	8		0	23	
>7 days before labor	7	12		19	0	
LMWH*	8	14	0.27	8	23	0.08
Anesthesia: epidural*	75	80	0.49	75	88	0.17
General	1	5	0.05	4	8	0.52
Pethidine	4	1	0.27	2	0	0.45
Ampicillin peripartum*	10	7	0.42	8	4	0.46
Hypothyroidism*	14	22	0.16	12	38	**0.01**
Smoking*	N/A	23	–	21	27	0.55
PIH/preeclampsia*	N/A	9	–	8	12	0.65
Thrombophilia*	N/A	7	–	8	4	0.49
HC (cm)	N/A	32.70 (31.50–34.00)	–	33.6 (32.5–34.3)	31.2 (29.7–32.0)	**<0.001**
AC (cm)	N/A	28.25 (26.10–29.50)	–	28.9 (27.5–30.5)	26.0 (25.0–27.5)	**<0.001**
CI	N/A	1.30 (1.24–1.51)	–	1.3 (1.2–1.3)	1.6 (1.5–1.8)	**<0.001**
HC/AC	N/A	1.15 (1.11–1.21)	–	1.1 (1.1–1.2)	1.2 (1.1–1.2)	0.26

Arithmetic data are shown as median values (25–75th pctl). ^*^Categorical characteristics are presented as percentages. Bold values indicate statistical significance. T, temperature; LMWH, low molecular weight heparin; PIH, pregnancy-induced hypertension; HC, head circumference; AC, abdominal circumference; CI, cephalization index; N/A, not available.

Blood was collected from the umbilical cord vein immediately after clamping the cord. All neonates (FGR and control group) underwent delayed cord clamping for 1 min after birth. The specimen was drawn smoothly with a 21-gauge needle into plastic tubes containing 3.2% (0.109 M) buffered sodium citrate (blood: citrate = 9:1) and stored at room temperature after gentle mixing with the anticoagulant. All samples were tested *via* PFA-100^®^–Platelet Function Analyzer (DADE BEHRING) within 4 h according to manufacturer's instructions as previously described (21, 22). PFA-100 is a cartridge system of *in vitro* assessment of primary, platelet-related hemostasis that is sensitive to different preanalytical factors such as hemostatic defects, medication effects, platelet deficiencies, and hematocrit disturbances (21, 23), which were taken into consideration in order to obtain more valuable results. Closure times (CTs) for both stimulating agents collagen and epinephrine (COL/EPI) or adenosine 5'-phosphate (COL/ADP) were determined. Additional tests included the complete blood count (CBC) (Abbott cell-dyn 3,700 hematology analyzer) and blood group. Platelet count was confirmed by microscopic evaluation of peripheral blood smears.

Statistical analysis was performed by SAS 9.4 for Windows (SAS Institute Inc., NC, USA) (24). For differences of data expressed in a numeric form, the Kruskal-Wallis test was performed, while comparisons or proportions of qualitative data (Normal/Abnormal or Yes/No values) were performed *via* the chi-square test. Odds ratios were evaluated *via* the Wald's *p*-value. The statistical significance level was set to 0.05 and all tests were two-tailed.

## Results

**Figure 2:**
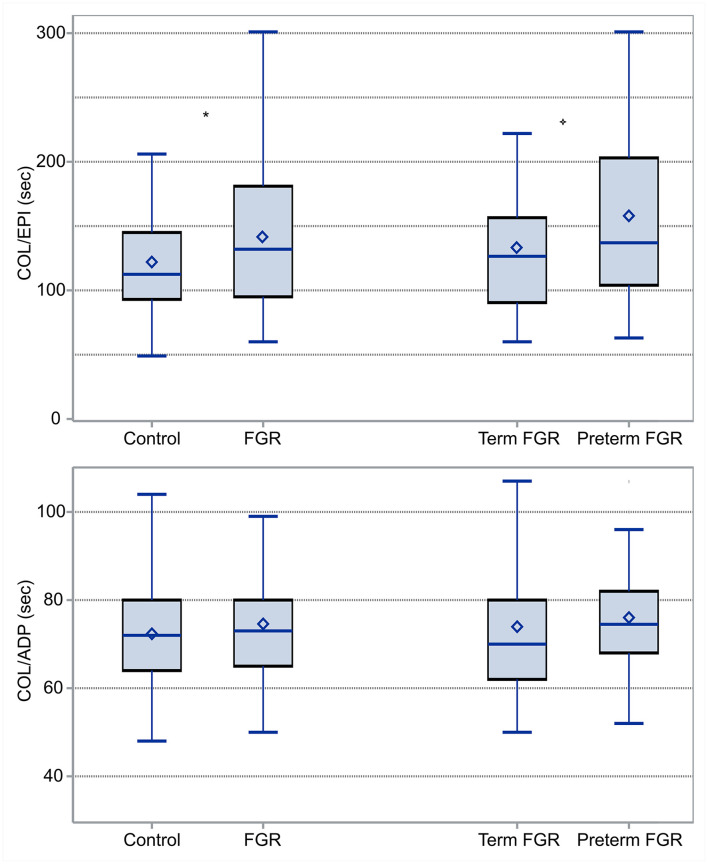
Box and Whisker plots comparing COL/EPI **(top)** and COL/ADP **(bottom)** CTs between FGR and control neonates and term and preterm FGR neonates: COL/EPI and COL/ADP are expressed in seconds. *indicates statistically significant difference.

COL/EPI and COL/ADP CTs were compared between FGR and AGA neonates (control group). COL/EPI CTs were prolonged in FGR neonates (median 132 s, IQR 95–181 s) compared with control neonates (median 112.5 s, IQR 93–145 s), *p* = 0.04. No differences were found for COL/ADP CTs, with median COL/ADP CTs of 73 s in FGR neonates (IQR 65–80 s) and 72 s in control neonates (IQR 64–80 s), *p* = 0.55, as shown on Figure 2. Further analysis between FGR and control neonates showed that FGR neonates were characterized by lower BW (*p* < 0.001), BW centile (*p* < 0.001), GA (*p* < 0.001), temperature at birth (*p* = 0.007), and Apgar score at 1st min (*p* = 0.04). A higher percentage of pregnant women of FGR fetuses received aspirin prophylaxis compared with control fetuses (*p* = 0.04). FGR neonates had lower median WBCs count (*p* < 0.001), higher Hb (*p* < 0.001), and higher Hct (*p* < 0.001).

Additionally, differences between term (*N* = 48) and preterm (*N* = 24) neonates among the group of FGR neonates were tested. According to our results, median COL/EPI CTs for term and preterm FGR neonates were 126 s (IQR 90–157 s) and 137 s (IQR 104–203 s), respectively, and accordingly, median COL/ADP CTs were 70 s (IQR 62–80 s) for term and 75 s (IQR 68–82 s) for preterm FGR neonates. Difference was found only in COL/EPI CT (*p* = 0.001) (Figure 2). Moreover, preterm FGR neonates had lower median BW (*p* < 0.001), BW centile (*p* < 0.001), Apgar score at 1st min (*p* = 0.02), median HC (*p* < 0.001), median AC (*p* < 0.001), and higher CI (*p* < 0.001) compared with term FGR neonates. A higher percentage of *in vitro* fertilization (IVF) (*p* = 0.03), caesarian section (CS) (*p* = 0.006), aspirin (*p* < 0.001), and levothyroxine administration (*p* = 0.01) were observed among mothers of preterm FGR neonates (Tables 1, 2).

A mixed model to control the effects of maternal aspirin administration on neonatal CTs was implemented. Results showed that aspirin administration had no effect on COL/EPI or COL/ADP CTs neither in the complete population, nor in the FGR or control group, nor when adjusting for term and preterm neonates (*p* > 0.05 in all cases). The same results were obtained when aspirin administration was grouped into <7 or >7 days from delivery. Furthermore, a multivariate linear regression model was applied to identify factors that could affect both CTs in the whole group of FGR neonates. The statistically important parameter for COL/EPI CT was delivery time (with preterm neonates exhibiting prolonged COL/EPI CTs, *p* = 0.05). As far as COL/ADP CT is concerned, no parameter showed statistical significance.

Finally, we evaluated COL/EPI and COL/ADP CTs in accordance with different parameters in the group of FGR neonates. COL/ADP CTs were shorter in FGR neonates born *via* vaginal delivery, compared with those born *via* CS [62 s (60–75 s) vs. 74 s (67–86 s), (*p* = 0.007)]. COL/ADP CTs were prolonged in FGR neonates whose mothers had received epidural anesthesia compared with other methods of anesthesia [73 s (67–86 s) vs. 62 s (59–75 s), (*p* = 0.03)]. COL/ADP CTs were shorter after intrapartum antibiotic prophylaxis (IAP) with ampicillin compared with no antibiotics [59 s (57–60 s) vs. 74 s (66–82 s), (*p* = 0.005)]. The specific cause of FGR seems to have no impact on CTs (*p* > 0.05 in all cases) (Supplementary Table 1). As expected, COL/EPI CTs and COL/ADP CTs had a positive correlation (*r* = 0.37, *p* < 0.001). No correlation was proved between COL/EPI and COL/ADP CTs and hematological parameters in FGR neonates.

## Discussion

Fetal growth restricted neonates confront a significant risk of perinatal morbidities, including hemostatic impairment, and thus, platelet-related primary hemostasis needs to be carefully evaluated. To the best of our knowledge, our cohort represents a study of platelet function *via* PFA-100 in the largest number of FGR neonates (*n* = 74) reported so far. To make reasonable interpretation, all data and laboratory findings were compared with a control group of 118 AGA neonates.

Lower BW and BW centile were reasonable according to the definition of FGR neonates. The higher rate of preterm births in FGR neonates is reasonable, as the risk of prematurity in FGR is 3-fold greater than in AGA infants (25). The well-known risk of FGR infants for irregular thermoregulation (26) explains the lower temperature observed in FGR group. FGR infants have a greater risk of perinatal stress due to a sentinel event superimposed on chronic fetal hypoxia from placental insufficiency (3) and this explains lower Apgar scores in FGR newborns, as mentioned in our study.

As expected, preterm neonates had lower somatometric parameters (BW, BW centile, AC, and HC). Furthermore, higher CI and lower Apgar scores reflect the greater compromise of prematurity. A higher percentage of preterm births was observed in pregnancies conceived by IVF, in accordance with the current literature (27). Although there is no consensus in the optimal delivery method for a preterm birth, even in FGR fetuses, our institution's practice favors CS as depicted with the higher percentage of preterm neonates born *via* CS (28, 29). The higher incidence of preterm labor in pregnant women with hypothyroidism, despite levothyroxine replacement therapy (30), supports higher percentage of preterm birth in our group of FGR neonates.

Regarding primary hemostasis, COL/EPI CTs were prolonged in FGR compared with control neonates, whereas no corresponding difference was found for COL/ADP CTs. According to the helpful algorithm proposed by Favaloro, prolonged COL/EPI CTs with normal COL/ADP CTs could be attributed to drug effect, low Hct, mild thrombocytopenia, and mild platelet/von Willebrand factor (VWF) dysfunction (31). Aspirin is well-known to prolong COL/EPI CT, when COL/ADP CT is usually normal (23). It is worth noticing that 20% of mothers of our FGR neonates received aspirin during pregnancy, in contrast to 8% of mothers of control newborns. This finding complies with the recommendation of low-dose aspirin prophylaxis, initiated before 16 weeks of gestation for women at a high risk of preeclampsia (32). Subanalysis and linear regression model analysis support that aspirin administration during pregnancy had no effect on COL/EPI or COL/ADP CTs, neither in the complete population nor in the FGR or control group. A small amount of acetylsalicylic acid reaches fetal circulation (33) and results in reduced levels of thromboxane B_2_ in the umbilical cord (34, 35) but, according to our findings and previous platelet aggregation studies, neonatal platelet function does not appear to be affected (36, 37).

It has been shown that lower Hct is correlated with prolongation of COL/EPI CTs (31), while higher neonatal Hct seems to explain shorter bleeding time (BT) and CTs compared with adults, partly by rheologic effects (38). FGR neonates in our study were characterized by higher Hb and Hct, as polycythemia is common in FGR neonates (up to 50%) (39), due to relative hypoxia (40) that likely triggers red cell production (41–44). Thus, the prolongation of COL/EPI CTs among FGR neonates of the present study could not be attributed to the Hct effect. In fact, we could assume that higher Hct of FGR neonates could not compensate impaired activation of platelets.

Low platelet counts were correlated with prolongation of CTs and should be taken into consideration when interpreting CTs (21, 45, 46). It is well-known that the incidence of thrombocytopenia in FGR neonates is higher and more prominent as the severity of FGR increases (7, 8). In our study, the median platelet count of the FGR group did not differ significantly from the corresponding of the control group, as cord blood samples with platelets lower than 100 × 10^9^/L were excluded according to our study design. Thus, thrombocytopenia was excluded as a confounding factor in interpreting CT values and the observed prolongation of COL/EPI CTs was attributed to platelet function abnormalities.

Prolongation of COL/EPI CTs of FGR neonates seems to be the result of platelet dysfunction, although this remains a field of further investigation for this group of neonates, as the majority of studies concerning activation of platelets in FGR neonates were conducted on the subpopulation of neonates born to mothers with PIH. A study of CPA reports lower platelet adhesion in FGR infants (13) and another study supports that preeclampsia influences the expression of GPs on activated neonatal platelet-surface, which may affect platelet function, leading to an additional bleeding risk in thrombocytopenic neonates (14). A study showed that platelets of neonates of preeclamptic mothers had a markedly higher responsiveness to agonists *in vitro* by flow cytometry; however, resting platelets of FGR neonates seemed to be in a slightly lower state of activation (47). Although the use of PFA as a screening tool for inherited platelet function disorders among adults has been questioned (48), prolongation of COL/EPI CT in the present study supports the impaired platelet function of FGR neonates reported from previous studies (13, 14, 47). It is of great importance that platelet hyporesponsiveness seems to apply for the whole group of FGR neonates irrespective of the cause.

As far as white blood cell count is concerned, neutropenia is frequent in offsprings of pregnancies complicated with PIH (43, 49). FGR neonates of our study were also characterized by lower median values of total leucocytes count compared with the control group. However, leucocytes do not have any influence on CTs (50, 51).

Regarding the role of prematurity in our FGR group, COL/EPI CTs of preterm FGR neonates were prolonged compared with term FGR ones. COL/ADP CTs of preterm FGR neonates were also prolonged but not significantly. Multivariate linear regression analysis supported that prematurity affects COL/EPI CTs, whereas no corresponding relation was found for COL/ADP CTs. Concerning low-dose aspirin administration, despite the fact that more preterm FGR neonates were exposed *in utero* to aspirin, our subanalysis supports that aspirin administration during pregnancy had no effect on COL/EPI or COL/ADP CTs as previously mentioned. Preterm and term FGR neonates did not differ in any hematological parameter (Table 2), so we could not speculate that differences in Hct or platelet count are responsible for the prolongation of COL/EPI CTs. Prematurity is a well-known factor affecting platelet responsiveness. Preterm neonates have prolonged bleeding time (BT) (52), decreased platelet reactivity on flow cytometry studies (53), decreased platelet adhesion on CPA (54, 55), prolonged COL/EPI CT (16) or prolonged COL/ADP CT (56) on PFA-100, and lower maximum clot firmness (MCF) on rotational thromboelastometry (ROTEM) (57). The available evidence suggests that the platelet hyporeactivity is less well-compensated by other factors, such as high Hct, in preterm compared with full-term neonates. Although preterm FGR neonates in this study group were mainly late preterm neonates (meaning GA 34–36 + 6 weeks), our results highlight that platelet dysfunction that accompanies prematurity also applies in FGR preterm neonates. This was also reported in offsprings of pregnancies complicated by PIH with evidence of lower platelet adhesion on CPA, denoting an impaired platelet function (13). It is worth noticing that no bleeding event was recorded in our study group, and this could be attributed to our study population, which did not include neonates with extreme or moderate prematurity (GA <33 weeks) and severe thrombocytopenia, two parameters that characterize FGR neonates with the greatest bleeding risk.

The observation that the difference in responsiveness between platelets of FGR and control neonates is apparent to some agonists (epinephrine) rather than others (ADP) raises several questions concerning neonatal platelet function. Previous studies have shown that COL/ADP CT exhibits a negative correlation with GA (56) and platelet count (58) and, according to a recent study, it was proposed as a tool for bleeding risk assessment (59). In our study, however, no differences were found for COL/ADP CTs in FGR compared with AGA neonates. A possible explanation could be an increase in the available erythrocyte ADP caused by the higher Hct in FGR neonates, which may compensate for the decreased ADP secretion from neonatal platelets (38). Furthermore, a flow cytometry study showed that platelets of neonates of preeclamptic mothers had markedly higher responsiveness to ADP as an agonist, after *in vitro* activation (47).

It is worth noticing that COL/ADP CTs were found to be shorter in FGR neonates delivered *via* vaginal delivery and this could be attributed to the stimulant effect of cytokines, as both IL-6 and IL-1β induce platelet activation and aggregation through several mechanisms (60–64). Higher levels of cytokines are expressed in cord blood samples of vaginal deliveries of term neonates (65–67) and possibly overexpressed in FGR (68) and preterm (69) deliveries.

As far as the role of anesthesia is concerned, COL/ADP CTs were prolonged in our group of FGR neonates whose mothers received epidural anesthesia during labor. Opioids seem to have no adverse effect on platelet activation and aggregation. In contrast, platelet-activating factor (PAF) concentrations decrease with lignocaine (70) and platelet aggregation is inhibited by ropivacaine *via* PFA-100 (71).

Although previous studies support that ampicillin inhibits ADP-induced platelet aggregation in adults (72, 73), VLBW neonates (74), and NICU patients (75) *via* several mechanisms (76), the opposite was reported in our study. A possible explanation could be the small number of neonates whose mothers received ampicillin peripartum (5 of 74). Moreover, FGR neonates of our study did not have any evidence of bacterial infection as opposed to neonates receiving ampicillin in previous studies (74, 75).

It is worth mentioning that vWF could have possibly contributed to the differences of CT measurements in PFA-100 assay (31). Although assessing vWF levels was beyond the scope of our study, as we aimed to use PFA-100 as a point-of-care tool, this could be considered as a limitation of our study. Moreover, all measurements were conducted on cord blood samples. Cord blood has been suggested for initial workup especially in preterm infants (77) and the majority of studies using PFA-100 in neonatal population were conducted on cord blood samples (22, 38, 50, 78, 79). Cord blood samples are reliable when assessing complete blood count, including platelets (77) and its membrane glycoproteins *via* flow cytometry (53). However, especially when it comes to bleeding risk, results from umbilical cord samples should be interpreted with caution (56, 80, 81). Matching cord blood measurements with venous samples in a larger study could further shed light on the unexplored field of neonatal platelet function during the first few hours of an infant's life.

## Conclusion

In summary, the present study supports that FGR neonates demonstrate a relatively hyporesponsive phenotype. This platelet hyporesponsiveness, which should be further evaluated in a larger number of subjects, is overexpressed in preterm FGR neonates. In clinical practice, the evaluation of cord blood CTs, although depicting mainly the intrauterine hemostatic environment, could have a prospective character; cord blood samples offer the advantage of collecting larger blood volumes and perform several tests (i.e., blood gases, blood group, PFA-100), avoiding frequent neonatal blood sampling, a leading cause of anemia of the newborn. An abnormal CT value in a high-risk neonate could warn for closer evaluation and proper management during the first days of their lives.

## Data availability statement

The raw data supporting the conclusions of this article will be made available by the authors, without undue reservation.

## Ethics statement

The studies involving human participants were reviewed and approved by Ethics Committee of Aretaieio Hospital, National and Kapodistrian University of Athens (31.01.2017). Written informed consent to participate in this study was provided by the participants' legal guardian/next of kin.

## Author contributions

MK collected and analyzed data, wrote the initial draft of manuscript, and reviewed and revised the manuscript. NI, SV, MP, and TB conceptualized and designed the study, coordinated and supervised data collection, analyzed data, revised the initial draft manuscript, and reviewed and revised the manuscript. ZI and RS critically reviewed the manuscript for important intellectual content. VM collected data and reviewed the manuscript. AP and MB carried out statistical analyses. All authors approved the final manuscript as submitted and agree to be accountable for all aspects of the work.

## Conflict of interest

The authors declare that the research was conducted in the absence of any commercial or financial relationships that could be construed as a potential conflict of interest.

## Publisher's note

All claims expressed in this article are solely those of the authors and do not necessarily represent those of their affiliated organizations, or those of the publisher, the editors and the reviewers. Any product that may be evaluated in this article, or claim that may be made by its manufacturer, is not guaranteed or endorsed by the publisher.

## Supplementary material

Supplementary Table 1 Neonatal COL/EPI and COL/ADP CTs values as median (IQR) with regard to the cause of FGR.

## Abbreviations

AC: abdominal circumference
ADP: adenosine diphosphate
AGA: appropriate for gestational age
BT: bleeding time
BW: birthweight
CBC: complete blood count
CI: cephalization index
COL: collagen
CPA: cone and platelet analyzer
CS: caesarian section
CT: closure time
EFW: estimated fetal weight
EPI: epinephrine
FGR: fetal growth restriction
GA: gestational age
HC: head circumference
IAP: intrapartum antibiotic prophylaxis
IVF: *in vitro* fertilization
MCF: maximum clot firmness
NICU: neonatal intensive care unit
PAF: platelet activating factor
PFA: platelet function analyzer
PI: pulsatility index
PIH: pregnancy induced hypertension
ROTEM: rotational thromboelastometry
TEG: thromboelastography
VLBW: very low birthweight
VWF: von willebrand factor.
