# Supplementary material for: Primary hemostasis in fetal growth restricted neonates studied via PFA-100 in cord blood samples
Source: Front Pediatr. 2022 Sep 8;10:946932. doi: 10.3389/fped.2022.946932 (PMC9492943; doi:10.3389/fped.2022.946932)
Supplement: Supplementary Table 1 — Neonatal COL/EPI and COL/ADP CTs values as median (IQR) with regard to the cause of FGR. [file Table_1.docx]

**Supplementary Table 1.** Neonatal COL/EPI and COL/ADP CTs values as Median (IQR) with regard to the cause of FGR.

| **FGR cause** |  | **COL/EPI CT (sec)** | **p value** | **COL/ADP CT (sec)** | **p value** |
| --- | --- | --- | --- | --- | --- |
| Thrombophilia | *No* | 132(94-182) | 0.62 | 73(65-82) | 0.39 |
|  | *Yes* | 156(106-177) |  | 74(60-76) |  |
| Hypothyroidism | *No* | 135(94-177) | 1.00 | 73(63-82) | 0.82 |
|  | *Yes* | 122(101-193) |  | 73(68-80) |  |
| Smoking | *No* | 132(98-184) | 0.45 | 73(66-84) | 0.49 |
|  | *Yes* | 118(93-161) |  | 73(64-77) |  |
| PIH/preeclampsia | *No* | 132(95-184) | 0.42 | 73(65-82) | 0.18 |
|  | *Yes* | 127(94-154) |  | 68(57-77) |  |

CT: closure time, PIH: pregnancy-induced hypertension
